# Failure Behavior of Damaged Reinforced Concrete Pipe Rehabilitated with Fiber-Reinforced Mortar Lining

**DOI:** 10.3390/ma18133130

**Published:** 2025-07-02

**Authors:** Jieyao Li, Chunliang He, Yingjie Wei, Haoliang Wu, Jiajie Liao, Shun Dong, Sheng Huang, Baosong Ma

**Affiliations:** 1 School of Civil Engineering, Sun Yat-sen University, Zhuhai 519082, Chinawuhliang5@mail.sysu.edu.cn (H.W.);; 2 Southern Marine Science and Engineering Guangdong Laboratory (Zhuhai), Zhuhai 519082, China; 3 China Three Gorges Corporation, Wuhan 430010, China

**Keywords:** reinforced concrete pipe, fiber-reinforced mortar, trenchless rehabilitation, three-edge-bearing test, interface failure

## Abstract

The spray-applied pipe lining (SAPL) method, extensively employed in the trenchless rehabilitation of reinforced concrete pipes (RCPs) due to its operational versatility, remains constrained by an incomplete understanding of the failure behavior of rehabilitated pipelines, thereby impeding optimal design strategies. This study proposes an analytical approach to evaluate the structural performance of pipes with fiber-reinforced mortar lining, with a particular focus on interface failure and its consequences. Two RCPs with an inner diameter of 1000 mm, repaired with 34 mm and 45 mm centrifugally sprayed fiber-reinforced mortar liners, were subjected to three-edge-bearing (TEB) tests. The elastic limit loads of the two pipes were 57% and 39% of their pre-rehabilitation conditions, while the ultimate loads were 45% and 69%. A thicker liner exhibits a greater susceptibility to interface failure, leading to wider cracks around the elastic stage during loading. Once the interface failure occurs, load redistribution allows the liner to resist further cracking and sustain higher capacity, demonstrating enhanced bearing performance. Critical factors influencing the failure process were analyzed to inform design optimization, revealing that improving the interface takes precedence, followed by thickness design.

## 1. Introduction

By the end of 2023, the total length of urban drainage pipelines in China had expanded to 952,483 km, with nearly 50% of these pipelines having surpassed a service life of more than a decade [1]. Furthermore, it is estimated that nearly 75% of the aging pipes retain a functional lifespan of just 15 years, a duration markedly below the expected standard [2]. A vast network of drainage systems is urgently in need of renovation and upgrading. Reinforced concrete pipes (RCPs) are predominantly employed in drainage systems due to their adaptability and low cost. Nowadays, a significant proportion of these RCPs exhibit deterioration, manifesting in issues such as leakage, rupture, and corrosion, and eventually leading to structural damage. Failures of RCPs can be categorized into longitudinal cracks and corrosion, influenced by mechanical loading [3], environmental degradation [4] and material fatigue [5]. Notably, longitudinal cracks often result in crown collapse, which poses substantial risks to economic efficiency and public safety [6]. Consequently, the repair and maintenance of cracked RCPs emerge as a critical priority.

As an emerging science and technology, trenchless pipeline rehabilitation has been widely used in buried pipeline repair due to its little destruction to environments [7]. Compared to other rehabilitation technologies, such as cured-in-place pipe (CIPP) and formed-in-place pipe (FIPP), the spray-applied pipe lining (SAPL) method demonstrates superior post-rehabilitation structural capacity, coupled with distinct advantages of operational convenience and flexibility [8]. SAPL entails uniformly coating the inner surface of a host pipe with binding materials via centrifugal or pressure spraying to create a lining, making it ideal for the structural rehabilitation of pipes exceeding 600 mm in diameter. While materials such as epoxy, polyurea or polyurethane can be used, fiber-reinforced mortar is more prevalent due to its high strength and corrosion resistance [9]. Despite its widespread application in culvert and pipe renewal, a unified theoretical framework and standardized structural design approach—particularly for determining liner thickness—remain underdeveloped [10].

The double-layer construction of the host pipe and liner exhibits intricate mechanical behavior under external loads. Currently, a comprehensive theoretical framework that precisely characterizes the failure process is lacking. Jackson et al. [11] proposed a design method aimed at preventing cracks in the liner and concrete pipe caused by bending moments, highlighting the composite behavior of the repaired structure. Detailed behaviors, such as interface failure, were not discussed. The interfacial interaction between the liner and host pipe has frequently been overlooked due to its complexity. The lined pipes are often regarded as two pipes, leading to overly conservative designs [12,13]. Conversely, McAlpine [14] and Shi [15] conducted a theoretical analysis to emphasize that the interfacial bonding is pivotal in assessing the post-rehabilitation performance, with its debonding identified as the primary failure mode. These theories necessitate validation through comprehensive experimentation.

The three-edge-bearing (TEB) test, an indirect method in the design of buried RCPs, serves as an effective approach to assess the bearing capacity and deflection of pipes before and after rehabilitation, aiding structural performance evaluation [16]. Moore et al. [17,18] conducted TEB tests on corrugated steel pipes rehabilitated with mortar liners, revealing that the steel pipes and mortar liners were partially bonded. He et al. [19] confirmed that mortar-lined RCPs experience interfacial damage during loading, as demonstrated through a preloading experiment. Makisha [20] investigated mortar-lined RCPs reinforced with an interfacial mesh frame, showing the significant impact of interface conditions on structural performance.

This study integrates experimental and theoretical analyses to elucidate the failure behavior and mechanisms of RCPs rehabilitated with fiber-reinforced mortar lining, with a particular emphasis on interface failure and the interaction between the host pipe and the liner. The investigation commenced with comprehensive material and interface tests to establish foundational properties. Subsequently, large-scale TEB tests were conducted on two pipe specimens with different liner thicknesses, providing insights into the failure progression and the impact of thickness variations. Through deterministic and uncertainty analyses, a new failure model and design method that accounts for interface failure are proposed.

## 2. Failure Process of Mortar-Lined RCPs

### 2.1. Analytical Model

**Figure 1:**
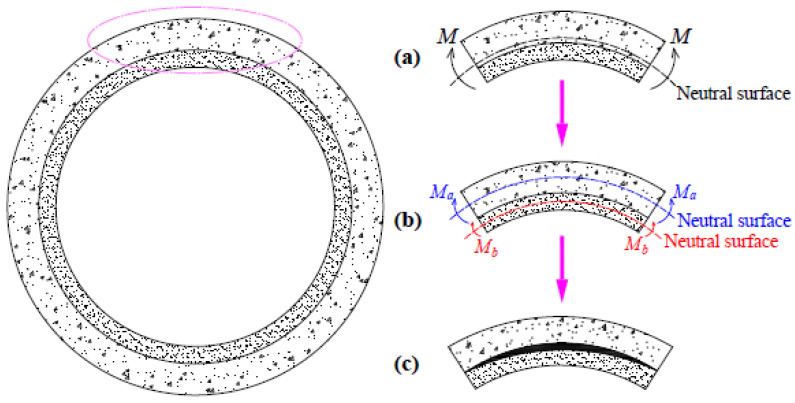
Analytical model of mortar-lined RCPs: (**a**) superposed curved beam (SCB); (**b**) composite curved beam (CCB); (**c**) two uncoordinated curved beams.

The mechanical properties of a pipe rehabilitated with mortar lining are influenced by the host pipe, the liner and their interface. The interface provides bonding that contributes to shear and tensile strength, allowing the host pipe and the liner to jointly bear external loads and undergo coordinated deformation. This configuration is regarded as a superposed structure. A part of the pipe section can be considered a curved beam, forming a superposed curved beam (SCB) after spraying, as shown in Figure 1a. If the condition of coordinated deformation is not achieved, the SCB degenerates into a composite curved beam (CCB) [21]. A composite beam refers to a beam where two materials are combined tightly but without adhesion at the interface, as shown in Figure 1b.

The SCB calculation method is advanced in this study, and a theoretical analysis of the CCB model is conducted. In the CCB model, it is assumed that differential deformations between the liner and the host pipe can be neglected, allowing them to undergo coordinated deformation. Upon pipe failure, the CCB transitions into two uncoordinated curved beams, with a distinct gap forming at the interface, as illustrated in Figure 1c. By employing deterministic and uncertainty analysis, this research will demonstrate the impact of interface on the failure behavior of the repaired pipe.

### 2.2. Interface Failure Criteria in SCB Model

**Figure 2:**
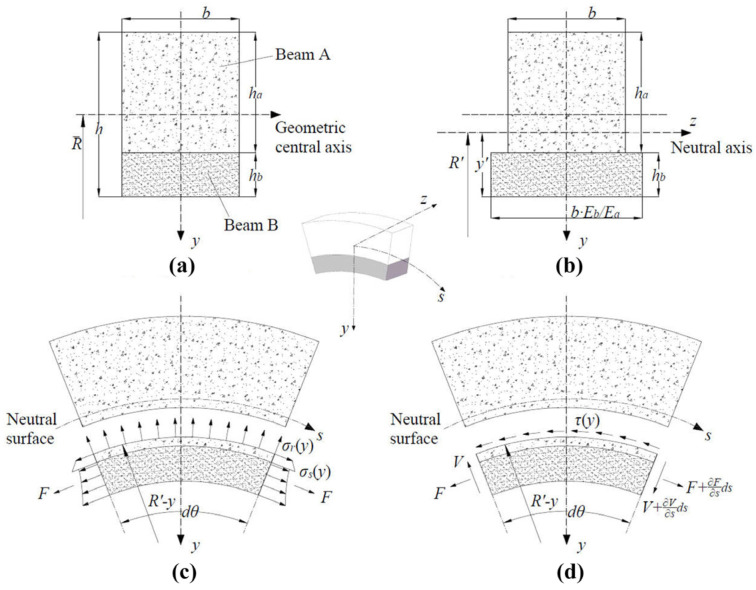
SCB model analysis: (**a**) original section; (**b**) transformed section; (**c**) normal and radial stress; (**d**) shear stress.

During elastic deformation, the reinforcement contributions in RCPs remain negligible. We consider a two-layer superposed curved beam consisting of beam A and beam B, which are bonded together. The original section is shown in Figure 2a. Referencing the variable section method [22], a transformed section (Figure 2b) is obtained. The distance of the neutral *z*-axis from the inner surface of beam B is calculated using Equation (1):

(1) $$y\prime =\frac{E_{a}h_{a}^{2}+2E_{a}h_{a}h_{b}+E_{b}h_{b}^{2}}{2E_{a}h_{a}+2E_{b}h_{b}}$$

where *E_a_* and *E_b_* are the elastic moduli of beam A and B, respectively; *h_a_* and *h_b_* are the heights of beam A and B, respectively.

Figure 2c,d illustrate an arc micro unit on the SCB. An element below is cut from a surface at any vertical position *y*. Similar to the derivation of a curved beam [23], the normal stress, radial stress and shear stress in beam B ($y\prime -h_{b}\leq y\leq y\prime$) are determined, as shown in Equations (2)–(4):

(2) $$\sigma_{s}(y)=\frac{E_{b}}{AD-BC}\left[DN-BM-(CN-AM)\cdot \frac{y}{1-\frac{y}{R\prime }}\right]$$

(3) $$\begin{array}{cc} \sigma_{r}(y) & =\frac{E_{b}}{\left(AD-BC)(R\prime -y\right)}[\left(DN-BM)(y\prime -y\right)\\ & \left.-(CN-AM)\cdot R\prime \left(R\prime \ln\frac{y\prime -R\prime }{y-R\prime }-y\prime +y)\right)\right] \end{array}$$

(4) $$\begin{array}{cc} \tau (y) & =\frac{V}{R\prime -y}\left\{\frac{E_{a}}{AD-BC}\left[(D-R\prime B)(y\prime -y)-(C-R\prime A)\cdot R\prime \left(R\prime \ln\frac{y\prime -R\prime }{y-R\prime }-y\prime +y)\right)\right]\right.\\ & \left.-\frac{E_{b}}{AG-C^{2}}\left[\frac{A}{6}y^{3}-\frac{C}{2}y^{2}-(\frac{A}{2}y\prime -C)y\prime \cdot y+(\frac{A}{3}y\prime -\frac{C}{2})y\prime^{2}\right]\right\} \end{array}$$

where the parameters are defined as follows:

(5) $$A=E_{a}A_{a}+E_{b}A_{b}$$

(6) $$B=E_{a}(\frac{J_{za}}{R\prime }+S_{za})+E_{b}(\frac{J_{zb}}{R\prime }+S_{zb})$$

(7) $$C=E_{a}S_{za}+E_{b}S_{zb}$$

(8) $$D=E_{a}J_{za}+E_{b}J_{zb}$$

(9) $$G=E_{a}I_{za}+E_{b}I_{zb}$$

(10) $$J_{z}=\int_{A}\frac{y^{2}}{1-\frac{y}{R\prime }}dA$$

where *M*, *N* and *V* are the bending moment, axial force and shear force, respectively; *Rʹ* is the radius of the SCB with respect to the neutral axis (*z*-axis); *A_a_* and *A_b_* are the sectional areas of beam A and B, respectively; *S_za_* and *S_zb_* are the static moments about the *z*-axis for beams A and B, respectively; and *I_za_* and *I_zb_* are the moments of inertia about the *z*-axis for beams A and B, respectively.

Therefore, interface failure can be evaluated by comparing the radial and shear stresses with the corresponding tensile and shear strengths at the interface. The criteria for maintaining the SCB are defined by Equation (11):

(11) $$\left\{\begin{array}{c} \left|\sigma_{r}(y\prime -h_{b})\right|\leq \left[\sigma_{r}\right]\\ \left|\tau (y\prime -h_{b})\right|\leq \left[\tau \right] \end{array}\right.$$

where [*σ_r_*] and [*τ*] are the interfacial tensile and shear strengths, respectively.

### 2.3. Uncertainty Analysis of Interface Failure

Considering that interface failure is strongly influenced by uncertainties, such as uneven thickness and variations in interfacial strengths due to inconsistent spraying quality, the failure probability analysis can be conducted. Assuming that the interface fails when at least one failure mode occurs, the failure probability is calculated using Equation (12). Monte Carlo simulation can be used to generate the probability distribution, which requires statistical parameters as inputs. The material parameters of concrete and mortar can be obtained from experimental data and usually follow normal distributions. The coefficients of variation for the size parameters influenced by pipe manufacture are evaluated based on JCSS [24], as shown in Equation (13).

(12) $$P_{f}=P(\min(\frac{\left[\sigma_{r}\right]}{\left|\sigma_{r}(y\prime -h_{b})\right|}-1,\;\frac{\left[\tau \right]}{\left|\tau (y\prime -h_{b})\right|}-1)<0)$$

(13) $$CV_{X}=\min(\frac{4+0.006X}{X},\frac{10}{X})$$

where *X* is the diameter, length or wall thickness (mm) of the RCP.

Moreover, as the failure criterion incorporates an excessive number of parameters, essential factors must be identified to inform engineering applications. To identify the primary factors influencing interface failure, sensitivity analysis can be performed. Sobol global sensitivity analysis, known for its comprehensive and explanatory nature in uncertainty quantification, employs a variance-based Monte Carlo sampling to decompose the output variance of a model into contributions from individual input variables and their interactions, quantifying each variable’s impact on the output [25]. First-order and second-order sensitivity indices quantify the contribution of a single input variable and the interaction between two variables to the output variance, respectively. The total sensitivity index accounts for the total contribution of an input variable to the output. For independently and identically distributed input variables *X*_1_, *X*_2_, …, *X_k_*, the model output is a function of these input variables, i.e., *Y* = *f*(*X*_1_, *X*_2_, …, *X_k_*). Each index is calculated as shown in Equations (14)–(16), and the sensitivity can be assessed by comparing the relative magnitudes of the indices for each input variable.

(14) $$S_{i}=\frac{V_{i}}{\mathrm{Var}(Y)}$$

(15) $$S_{ij}=\frac{V_{ij}}{\mathrm{Var}(Y)}$$

(16) $$S_{Ti}=\frac{V_{i}+\sum_{j\neq i}V_{ij}+\ldots }{\mathrm{Var}(Y)}$$

where *V_i_* is the individual variance contribution of *X_i_*, $V_{i}=\mathrm{Var}(E[Y|X_{i}])$; *V_ij_* is the contribution of the interaction between *X_i_* and *X_j_* to the output variance, $V_{ij}=\mathrm{Var}(E[Y|X_{i},X_{j}])-V_{i}-V_{j}$.

### 2.4. Load Redistribution in CCB Models

After interface failure, the SCB model degenerates into the CCB model. At this stage, the liner and host pipe no longer share the external load equally but instead bear separate portions of the load, leading to load redistribution. Since coordinated deformation is assumed, the load distribution can be derived based on the deformation condition, which is related to the stiffness and radius of the RCP. Referring to the method of deriving the loading capacity in slip-lining rehabilitation [26], the respective load on the host pipe and liner is calculated as follows:

(17) $$\frac{P_{a}}{\frac{B_{s}}{r_{a}^{3}}}=\frac{P_{b}}{\frac{E_{b}I_{b}}{r_{b}^{3}}}$$

(18) $$P_{a}+P_{b}=P$$

where *P_a_* and *P_b_* are the loads on the host pipe and liner, respectively; *P* is the total load; *r_a_* and *r_b_* are the radii of the host pipe and liner, respectively; *I_b_* is the inertia moment of the liner; and *B_s_* is the short-term stiffness of reinforced concrete, which is calculated by the following formula, according to GB/T 50010-2010 [27]:

(19) $$B_{S}=\frac{E_{s}A_{s}h_{0}^{2}}{1.15\psi +0.2+\frac{6\alpha_{E}\rho }{1+3.5\gamma_{f}^{\prime }}}$$

(20) $$\psi =1.1-\frac{0.65f_{tk}}{\rho_{te}\sigma_{sq}}$$

where *E_s_* is the elastic modulus of the rebar; *A_s_* is the area of tensile rebars; *h*_0_ is the effective thickness of host pipe; *ψ* is the nonuniform coefficient of strains; *α_E_* is the ratio of the elastic modulus of rebar to the elastic modulus of concrete; *ρ* is the reinforcement percentage of tensile rebars; *γ_f_*′ is the ratio of the effective area of the compressed flange to that of the web in a T-section (*γ_f_*′ = 0 for a rectangular section); *f_tk_* is the characteristic value of concrete tensile strength; *ρ_te_* is the effective reinforcement percentage; and *σ_sq_* is the tensile stress of rebars at the crack section.

After interface failure, the liner behaves as a single curved beam, and its normal stress can be calculated using the following formula:

(21) $$\sigma_{sb}(y_{1})=\frac{N_{b}}{A_{b}}-\frac{M_{b}}{A_{b}r_{b}}+\frac{M_{b}}{J_{b}}\cdot \frac{y_{1}}{1-\frac{y_{1}}{r_{b}}},\;-\frac{h_{b}}{2}\leq y_{1}\leq \frac{h_{b}}{2}$$

where *N_b_* and *M_b_* are the axial force and bending moment of the liner, respectively, calculated using *P_b_*; *A_b_* is the sectional area; and *J_b_* is calculated using Equation (10), with respect to the neutral axis of the liner.

## 3. Experimental Design

### 3.1. Pipe Specimens and Materials

Two RCP specimens with an internal diameter of 1000 mm (DN1000) were manufactured according to GB/T 11836-2023 [28], labeled as A and B. These specimens were classified as Grade II pipes as per the specification, with standard crack loads determined based on a crack width threshold of 0.2 mm [29]. The pipes were constructed using C50 concrete, and the reinforcement cages were composed of CRB550 rebars. Detailed parameters are presented in Table 1.

ConLining H70 mortar, a high-performance spraying material developed by Wuhan CUG Trenchless Technology Research Institute (Wuhan, China), was utilized. It is a composite material primarily composed of ordinary silicate cement and supplementary inorganic cementitious components, reinforced with fibrous elements, quartz sand aggregates and performance-enhancing additives. It is extensively employed in the rehabilitation of buried pipelines, whose technical specifications can be referenced in the related study [30].

### 3.2. Material and Interface Tests

Mechanical properties of the concrete and mortar were determined through standard tests for cementitious materials. Concrete–mortar interface strength, a critical factor in evaluating interface failure, should be assessed through dedicated interface tests. Although there is a lack of relevant standards, many scholars have summarized the types and methods of interface testing [31,32].

**Figure 3:**
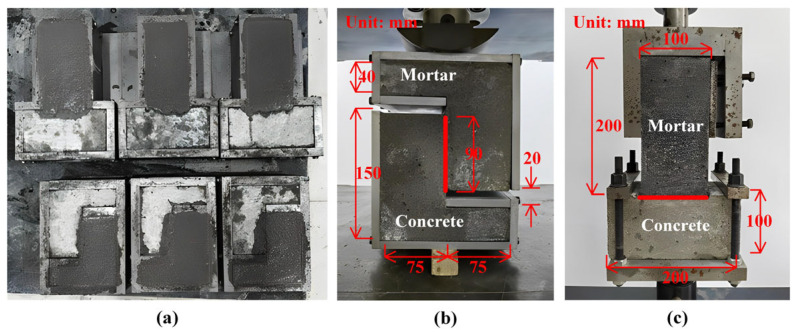
Interface tests: (**a**) specimen preparation; (**b**) shear test (thickness: 150 mm); (**c**) pull-out test (thickness: 100 mm).

To determine the interfacial shear and tensile strengths, direct shear tests and pull-out tests were performed, as illustrated in Figure 3. Z-shaped specimens were used in the shear tests, which was convenient for uniform loading and could reduce the influence of the bending moment [33]. The axial drawing method with T-shaped specimens was employed in the pull-out tests, which could reduce the influence of the loading deviation and stress concentration [34].

In a shear test, a steel plate ring was fixed around the periphery of the specimen to ensure that failure occurred along the interface. The universal testing machine by SUNS (Shenzhen, China) pressurized the specimen at 0.05 mm/s; during a pull-out test, the loading speed remained the same but reversed in direction. The testing machine continued loading until the specimens failed. The interface strength in each test was calculated as the ratio of the failure load to the interface contact area. Each test was performed on three specimens.

### 3.3. Equipment and Procedure of TEB Tests

**Figure 4:**
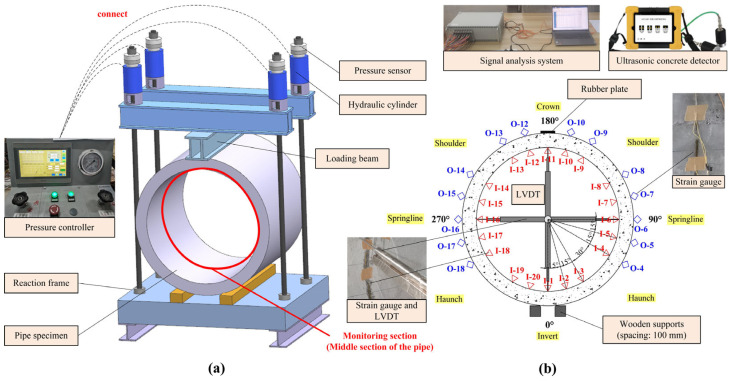
Arrangement of equipment: (**a**) loading system; (**b**) monitoring and data acquisition system.

The TEB tests were carried out in accordance with GB/T 16752-2017 [35]. A suite of experimental instruments was utilized, encompassing a loading system, displacement and strain monitoring systems and a data acquisition system. The hydraulic loading system, equipped with load sensors, was capable of exerting a maximum force over 100 ton on the pipes. A W-DCS50 LVDT displacement sensors by Beijing TYHC (Beijing, China) were used, with a measuring range of −50~50 mm. The strain gauge used for concrete strain measurement was BQ120-80AA by ZEMIC (Xian, China). Data collection was managed by the DH5921B signal analysis system from DONGHUA (Jingjiang, China). An HC-U81 ultrasonic tester by HICHANCE (Beijing, China) was employed to record the cracks and measure their widths, with a sampling period of 0.025~2000 ms. Mortar spraying was facilitated by the M/P-50 system from Wuhan CUG Trenchless Technology Research Institute (Wuhan, China). The placement of the equipment is shown in Figure 4. From the invert (0°), a strain gauge was set every 15° along the circumferential direction, except at the shoulders and haunches.

**Figure 5:**
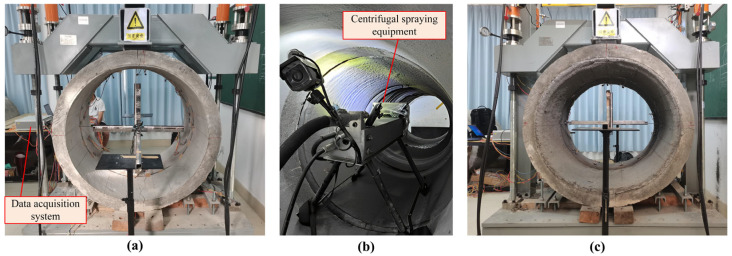
Pre- and post-rehabilitated RCP testing process: (**a**) host pipe being loaded; (**b**) mortar spraying; (**c**) repaired pipe being loaded.

The following procedure was employed to test pipe specimens A and B and their repaired counterparts:

- Before a test began, the internal and external surfaces of the monitoring section were cleaned, and the monitoring systems were set up.
- The hydraulic loading system was activated, and load was applied synchronously using four cylinders. The force discrepancy between each cylinder was consistently controlled to remain within a minor margin.
- Graded loading was applied, with each increment ranging from 5 to 6 kN over 1 to 3 min. During the loading process, cracks were monitored and measured. The system continued loading until the ultimate load was reached.
- After unloading the damaged pipe, its shape was restored. It was then rehabilitated with a centrifugally sprayed mortar lining and cured for 28 d.
- The actual thickness of the mortar lining was measured at the same locations as the strain gauges. Finally, the steps described above were repeated to test the repaired pipe. The testing procedure is shown in Figure 5.

### 3.4. Internal Forces of Pipes

When four hydraulic cylinders are loading simultaneously, the external load *P* on the pipe per unit length is calculated using Equation (22):

(22) $$P=\frac{\sum_{i}^{4}F_{i}}{L}$$

where *F_i_* is the load of each cylinder, and *L* is the length of the pipe.

Buried pipelines are predominantly damaged by bending moments, though other internal forces at critical cross-sections (crown, invert and springlines) also play a significant role, as effectively demonstrated by the TEB test. Since the net distance between the two wooden supports is 1/12 of the outer diameter of the pipe, the corresponding central angle is approximately 9°. Therefore, the loads can be simplified to bidirectional compressive loads at the crown and invert. The internal forces can be calculated using the following equations:

(23) $$M=\frac{PR}{\pi }(1-\frac{\pi }{2}sin\theta )$$

(24) $$N=-\frac{P}{2}\sin\theta$$

(25) $$V=-\frac{P}{2}\cos\theta$$

where *M*, *N* and *V* are the bending moment, axial force and shear force; *R* is the average radius of the circular ring; and *θ* is the angle measured anti-clockwise from the invert to the calculated cross-section.

## 4. Results and Discussion

### 4.1. Material and Interface Properties

The mechanical properties of the concrete and mortar were determined through standardized tests, with their average values and coefficients of variation provided in Table 2. Owing to the incorporation of reinforced fibers, the high-performance mortar exhibited significantly enhanced properties compared to conventional cementitious materials.

The interface test results reveal that the interfacial shear and tensile strengths are 1.44 (2.08%) and 0.72 (10.85%) MPa, respectively. All specimens exhibit damage along the interface, characterized by relatively smooth fracture surfaces. A small amount of fiber-reinforced mortar adheres to the concrete substrates.

### 4.2. Displacement and Deformation Responses

**Figure 6:**
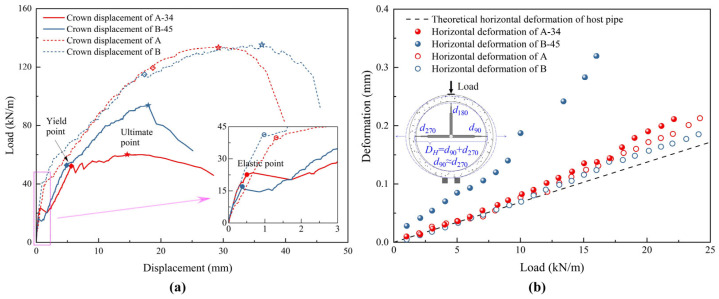
Displacement and deformation responses of pre- and post-rehabilitated RCPs: (**a**) load–displacement relationship; (**b**) deformation–load relationship.

The average spray thicknesses for specimens A and B are 34 (9.21%) and 45 (18.23%) mm. As the spray thickness increases, the uniformity of the liner thickness decreases due to challenges in the spraying process and the effects of gravity, which introduce an inherent systematic error. The repaired pipes are labeled as A-34 and B-45. The load-displacement response at the crown of each pipe is shown in Figure 6a. Table 3 presents the specific values reflecting the structure performance.

The overall structural performance of the repaired pipes is inferior to that of the pre-rehabilitation condition. The elastic limit loads of the repaired pipes are 57% and 39% of the pre-rehabilitated pipes, respectively, while their ultimate loads are 45% and 69%. During the elastic stage, the performance of the repaired structure primarily depends on the properties of the mortar, concrete, and their interaction. A pipe with a thicker lining exhibits a lower elastic limit load, which is related to the mechanical behavior of the interface. The yield loads for A-34 and B-45 are similar, influenced by the reinforcement in the RCPs. Since the reinforcement may have been partially damaged during the first loading, the yield loads of the repaired pipes are approximately reduced by 55%. After yielding, the bending stiffness of the RCP decreases, and the liner thickness has a more pronounced effect on the load-carrying capacity. A thicker liner strengthens the structure and increases the ultimate load. In general, increasing the liner thickness enhances the ultimate bearing capacity, but it also results in a reduction in the elastic limit load. Additionally, the failure condition of the host pipe affects the structural performance of the repaired pipe.

The measurements of specimen A indicate minimal displacement at the invert, with a displacement of only 0.19 mm at failure, which is 0.66% of the crown displacement. Consequently, the invert displacement can be neglected. In the TEB test, the horizontal deformation at the springlines, which is less influenced by the loading form than vertical deformation, can be derived using the theory of elastic stability [40], as shown in Equation (26):

(26) $$D_{H}=\frac{PR^{3}\cdot \left(1-v^{2}\right)}{2EI}\left(\frac{4}{\pi }-1\right)$$

where *EI* is the stiffness per unit length, and *v* is Poisson’s ratio of the cementitious material (*v* = 0.2 according to GB/T 50010-2010 [27]).

Figure 6b presents the deformation–load relationships of the pipes, showing that the deformation behavior of each pipe around the elastic phase conforms to the theoretical linear relationship. The propagation of micro-cracks and the weakening of the concrete–mortar interface cause the curves to gradually deviate from linearity. The accurate stiffness of the materials can be obtained by fitting the deformation using Equation (26), as shown in Table 3. The fitting effects are good, and the range of R^2^ is within 0.98~0.99. The stiffness of the repaired pipes is greater than that of the host pipes.

### 4.3. Damage Pattern and Strain Variation

**Figure 7:**
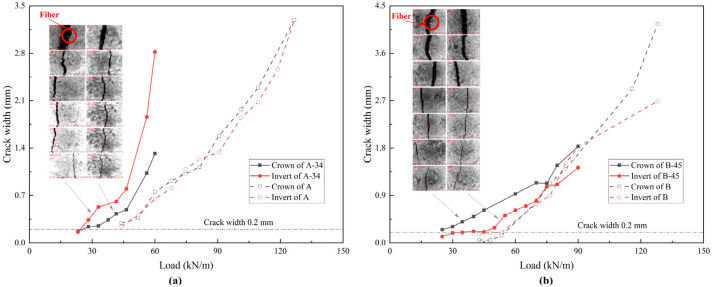
Development of crack width: (**a**) A and A-34; (**b**) B and B-45.

The cracks at the crowns and inverts were monitored and measured, with their propagation shown in Figure 7. Due to the influence of interface randomness, the liner cracks around the crown and invert have different development trends. For A and B, a crack with a width of 0.2 mm corresponds to the elastic limit load. However, the crack load is different for the repaired pipes: approximately 28 kN/m for A-34 and 22 kN/m for B-45, which misalign with their elastic limit loads. Thus, the elastic behavior of the repaired pipe is influenced predominately by the interfacial mechanical properties rather than by cracking. The fibers in the mortar inhibit cracking, resulting in narrower cracks than those in the concrete at the final stage.

**Figure 8:**
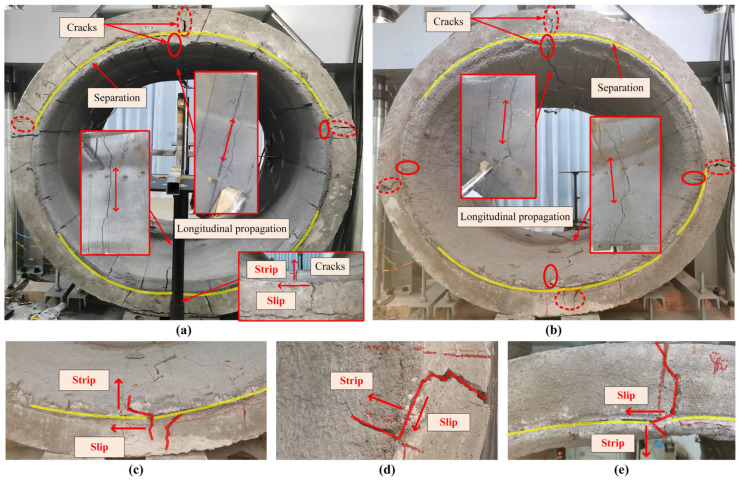
Damage condition: (**a**) A-34; (**b**) B-45; (**c**) invert of B-45; (**d**) springline (90°) of B-45; (**e**) crown of B-45.

Figure 8 illustrates the post-failure conditions of A-34 and B-45. Longitudinal cracks emerge on the inner surfaces of the lining around the crown and invert, whereas cracks at the springlines, originating at the interface, fail to penetrate the liner. Additionally, the damage includes separation between the host pipe and the liner. Due to gravity, separation is more pronounced on the upper side of the springlines than on the lower side. This kind of separation indicates two forms of interface failure: stripping and slipping. Stripping causes the detachment of the liner, while slippage induces misalignment between the cracks in the host pipe and those in the liner.

**Figure 9:**
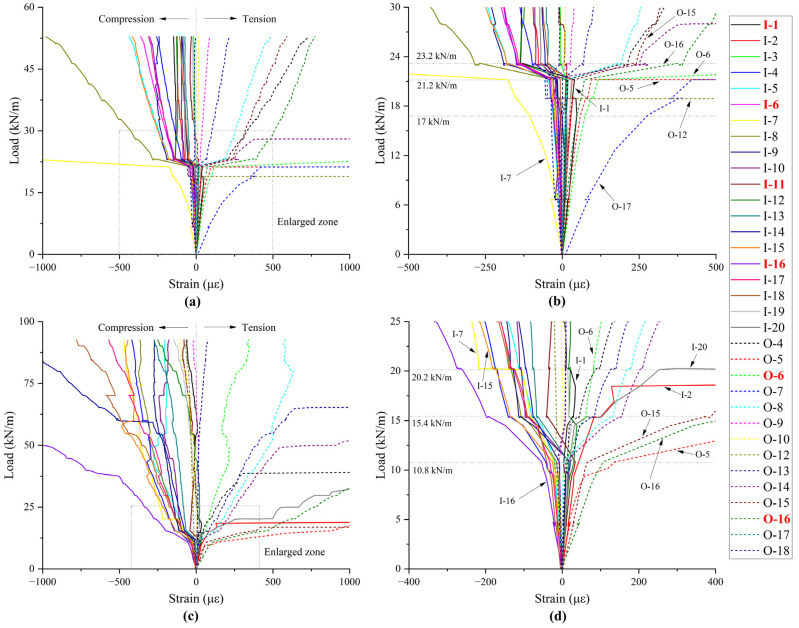
Strain variations: (**a**) A-34; (**b**) enlarged zone of A-34; (**c**) B-45; (**d**) enlarged zone of B-45.

The strain variations of the repaired pipes are presented in Figure 9, with notably anomalous values excluded. Strain values at and around the critical cross-sections are highlighted. The inner surface of the liner primarily experiences compression, while the outer surface of the host pipe is predominantly subjected to tension. Around the crown and invert, this situation is reversed. When a tension crack intersects a measuring point, the strain gauge becomes damaged. Inflections in the strain curve correspond to cracking or interface failure. The strain response of the rehabilitated pipe parallels that of its pre-rehabilitated counterpart for the thin liner. In contrast, the thicker liner markedly alters the strain behavior, highlighting the influence of interfacial interactions. The elastic limit point on the load-displacement curve is a comprehensive reflection of these inflection points. As shown in Figure 9b,d, the inflection points exhibit spatial asymmetry and randomness but align with specific phases corresponding to distinct load levels. This behavior is closely associated with the interface failure process of the superposed structure.

### 4.4. Interface Failure Behavior

**Figure 10:**
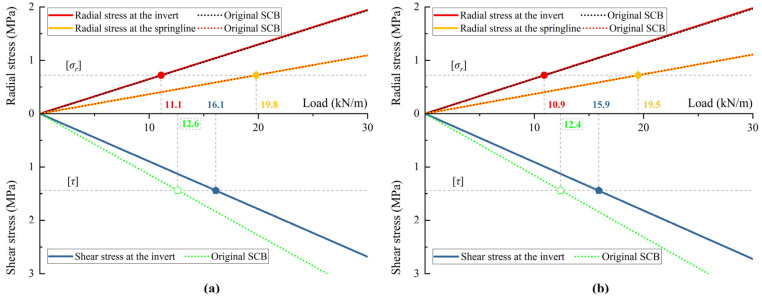
Interfacial stresses (compared with the original SCB model from Zhao et al. [21]) in critical cross-sections: (**a**) A-34; (**b**) B-35.

According to the advanced SCB model, the interfacial stresses at critical cross-sections are calculated using experimental data and compared with the theoretical predictions of Zhao et al. [21], as shown in Figure 10. Gravity is not accounted for in the theoretical calculations, resulting in identical stresses at the crown and invert. According to the bidirectional compression model, there is no shear at the springlines. The difference in interfacial radial stress between the two theories can be negligible, whereas the difference in shear stress is significant. As shown in Figure 9, the interfacial shear stress failure loads predicted by the proposed theory are closer to the load values corresponding to the inflection points in the strain curves (17 and 15.4 kN/m), showing more accurate calculation results.

At the invert, the interfacial radial stress first reaches the tensile strength, followed by the shear stress reaching the shear strength as the load increases. Therefore, the interface failure behavior can be summarized as follows: the interface at the invert and crown strips first, then slips, and finally, the interface at the springline strips. The interface failure load can be classified into interface stripping load and interface slipping load. It is important to note that interface failure refers to debonding rather than separation between the liner and the host pipe. Separation only occurs when the pipe is damaged to a certain extent, resulting in gaps at the interface. However, the direction of separation aligns with the direction of interfacial stresses.

As the liner thickness increases, the interface fails earlier, although the effect of thickness is minimal. When the thickness increases by about 10 mm, the interface failure load decreases by 200~300 N/m. The influence of interface failure is difficult to detect when the thickness is small. As shown in Figure 9, the strain curves of A-34 do not reflect the stripping at the invert. In contrast, the inflection points of B-45 clearly reflect the stripping and slipping processes of the interface. Overall, the interface bonding effect weakens with increasing liner thickness, as a thicker liner increases the deviation of the neutral axis during loading, resulting in higher stresses at the interface.

**Figure 11:**
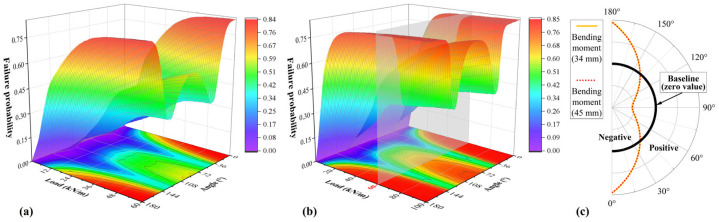
Interface failure probability: (**a**) A-34; (**b**) B-35; (**c**) typical bending moment distributions under the same load.

One million Monte Carlo simulations were performed for both A-34 and B-45. The relationship between interface failure probability, load and location is shown in Figure 11. The distribution of interface failure probability resembles the bending moment distribution, showing two peaks and valleys as the angle changes. The probability of interface failure is minimized at locations where the bending moment is zero and only axial and shear forces act. Under the same load, the effect of different liner thicknesses on the bending moment is minimal, resulting in a small influence of thickness on the interface in deterministic analysis. At approximately 12 kN/m, the failure probability at the crown/invert reaches 50% and increases rapidly thereafter. Once the ultimate load is reached, the certainty of interface failure becomes nearly absolute. The rate of increase in interface failure probability is faster for thicker liners up to 60 kN/m. Therefore, although spray thickness has a small effect on failure, uneven thickness can significantly increase the failure probability. Designing a thicker liner may lead to difficulties in spraying operations, resulting in an uneven distribution of thickness.

**Figure 12:**
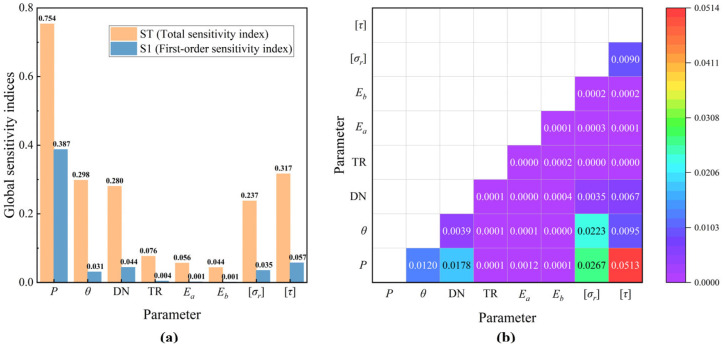
Sobol sensitivity indices of influencing parameters in failure criteria: (**a**) ST and S1 sensitivity indices; (**b**) S2 sensitivity index.

The SAPL rehabilitation method is suitable for DN600 and larger drainage pipes. According to GB/T 11836-2023, each pipe diameter corresponds to a minimum design wall thickness, resulting in discrete values for the dimensional parameters of the original RCP. To ensure water flow capacity, the spray thickness should not exceed the wall thickness of the host pipe. TR is used to express the thickness ratio of the liner to the pipe wall. The strength class of RCP concrete ranges from C25 to C60, while the strength of the lining mortar is selected based on high-performance concrete. When high-performance cementitious material is used to reinforce the old concrete with interface treatment, the upper limit of interface strength can reach up to 5 MPa [41,42]. Considering these factors, the sensitivities of the interface failure parameters are presented in Figure 12 after Monte Carlo sampling and calculation.

**Figure 13:**
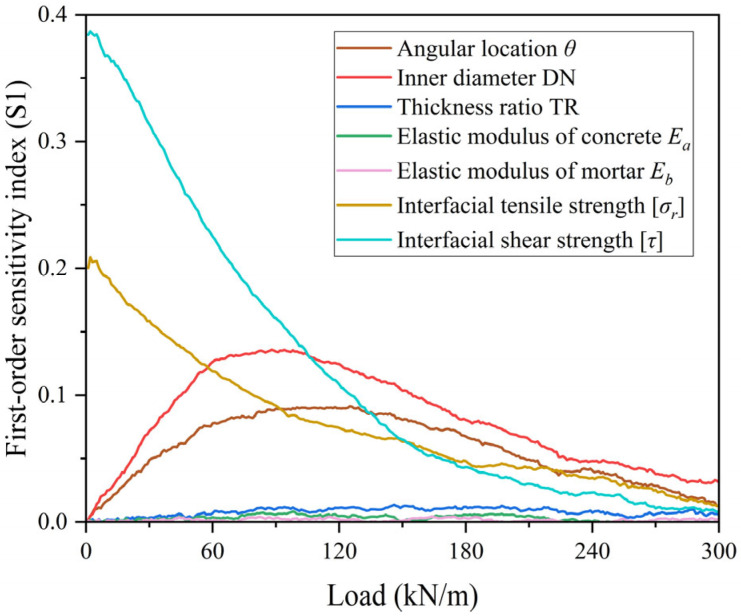
S1 sensitivity index under different load values.

Bending moments caused by external loads are the primary cause of interface failure, the magnitude of which depends on pipe diameter and spatial positioning. Among the material properties, the interfacial strength is the most critical, with its sensitivity significantly higher than that of thickness and elastic modulus. The elastic moduli have the least effect on interface failure, allowing the reduction in elastic modulus due to the cracking of the host pipe to be neglected. Further analysis reveals that the sensitivity of interface strength to pipe diameter and angle varies with loading conditions, as shown in Figure 13. In general, interfacial strengths, particularly shear strength, play a dominant role. Their effect decreases with increasing load, while the sensitivity to diameter and angle exhibits a maximum value. For a larger diameter RCP, the interface failure load increases, and the influence of interfacial strength on failure becomes less significant. Only at very high loads does the sensitivity of wall thickness and elastic modulus narrow the gap with other parameters. Efforts should focus on increasing the interfacial strengths to prevent interface failure.

### 4.5. Result of Interface Failure

**Figure 14:**
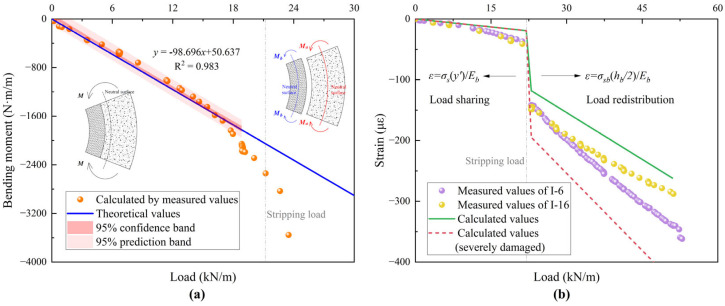
Validation of load redistribution in A-34: (**a**) bending moment at springline (90°); (**b**) strain on the internal surface at both springlines.

Upon debonding between the host pipe and the liner, each structural component independently resists external loads. To validate the load redistribution process, it must first be demonstrated that the liner and host pipe jointly bear the external load before interface failure. The measured bending moment is calculated using internal and external strain values, as expressed in Equation (27). Verification is directly achieved by comparing the measured bending moment with the theoretical one. For this analysis, strain values at the 90° position of A-34 are used, and the results are presented in Figure 14a. Before the interface at the springline strips, the theoretical and measured bending moments show good agreement.

(27) $$M_{measure}=EI\cdot \frac{\varepsilon_{in}-\varepsilon_{out}}{h_{a}+h_{b}}$$

where *ε_in_* and *ε_out_* are the internal and external wall strains, respectively.

As the load redistribution process begins, the measured values start to deviate from the theory. At this juncture, the strain of internal wall is employed for indirect comparative analysis. Load redistribution is governed predominantly by the stiffness of each component. Assuming the concrete can no longer resist tension, the short-term stiffness remains constant irrespective of the bending moment. Design document [28] shows that the short-term stiffness is about 2.09% of the original stiffness when buried RCP is severely damaged but has not collapsed. For the liner, its stiffness can be assumed to remain unchanged due to the high-performance mortar. The strain on the inner surface at the springline of A-34 is calculated and compared with the measured values, as shown in Figure 14b. The theoretical strain variation trend aligns well with the measured values, and the magnitudes are close, confirming the correctness of the load redistribution law. At the point of load redistribution, the liner strain changes abruptly. In reality, the liner withstands a greater load according to the measured values, indicating that the short-term stiffness of the host pipe becomes slightly smaller due to the first loading.

If the load exceeds the interface failure load at the cross-section, it is redistributed, leading to a sudden increase in stress in the liner. This makes the liner more prone to cracking. For the liner of a rigid pipe, the development of crack width at the crown/invert is related to the pipe deformation by the following equation [43]:

(28) $$w=t(\frac{1}{r}-\frac{1}{r’})=t\times \frac{M}{E_{b}I_{b}}$$

where *t* is the liner thickness; *r* and *r*′ are the radii of the liner before and after deformation, respectively.

**Figure 15:**
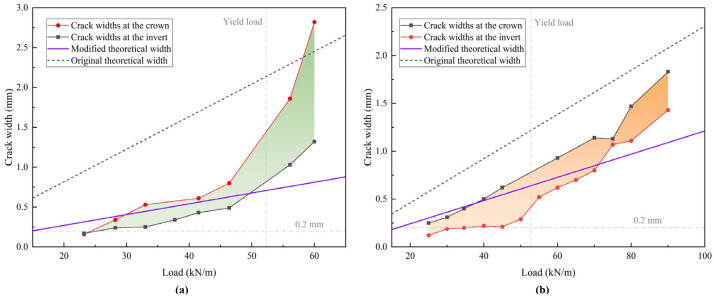
Theoretical development of crack width: (**a**) A-34; (**b**) B-45.

In the original theory, the change in curvature of the liner is caused by the total bending moment. This method calculates a much larger crack width than the actual situation, as shown in Figure 15, because it does not account for load redistribution. However, the liner bears only a portion of the bending moment *M_b_*, and the crack width is much smaller. The crack width calculated by the modified method lies within the measured values and matches the actual trend within the elastic–plastic phase. When the load exceeds the yield load, the calculated value is smaller due to the faster decrease in the stiffness of the host pipe. According to the principle of load redistribution, the liner bears a larger portion of the load, which accelerates cracking. In the short period before and after the redistribution, the thicker liner experiences more severe cracking due to interface failure. However, because of its greater stiffness, the extent of cracking in the thicker liner quickly becomes less than that in the thinner liner. In the post-yield plastic phase, the crack width in the thicker liner is smaller than that in the thinner liner, thus providing the repaired pipe with a higher bearing capacity.

**Figure 16:**
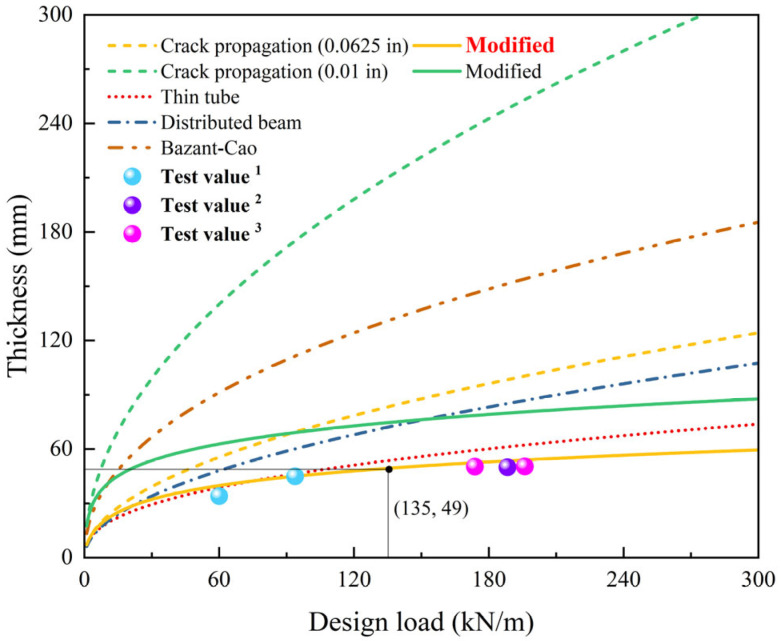
Comparison of design models for DN1000 RCP rehabilitated with mortar liners. (The test values 1, 2 and 3 in the figure are from: this paper, Zhao et al. [21] and He et al. [19], respectively.)

At the last stage, the repaired pipe becomes a “pipe-in-pipe” structure with almost global failure at its interface. The liner and the host pipe carry separate loads, and the bearing capacity of the repaired pipe depends on their individual capacities. Generally, the liner can no longer carry the load when cracking is severe, at which point the host RCP may not have reached plastic failure yet. In limit state design, the residual bearing capacity of the host pipe, which is mainly influenced by pre-rehabilitation load, becomes the determining factor. However, this parameter remains difficult to quantify. Therefore, the bearing capacity of the mortar lining is typically adopted as the design criterion, with spray thicknesses determined accordingly. Royer et al. [44] summarized commonly used design models, including those based on crack width, as shown in Table 4. However, the crack propagation model relies on the original theory of crack width, without considering the influence of interface failure. Based on the result of load redistribution, the external load is discounted, and the crack propagation model is modified in this paper. The comparison of these design models with the tested ultimate loads is illustrated in Figure 16.

Among the existing design models, the thin tube model is the closest to the actual situation. The crack propagation model with a design width of 0.01 in and the Bazant–Cao scaling models are very conservative and almost unusable in practice. The modified crack propagation model is closer to the test values, particularly the model with a design width of 0.0625 in, which is more realistic than the thin tube model. This is because the crack of 0.0625 in is very close to the crack width under ultimate load. When the wall thickness is approximately 49 mm, the repaired pipe can reach the bearing capacity of the pre-rehabilitated RCP. The modified model requires iterative calculations. In practical design, the external load can be multiplied by a reduction factor based on experimental data, or the safety factor can be appropriately reduced to save cost.

### 4.6. Discussion on the Entire Failure Process

The complete failure behavior of mortar-lined pipes is elucidated through deterministic analysis using the SCB and CCB models, leading to the proposal of an improved lining thickness design theory based on ultimate load. Theoretical predictions are validated against experimental values reflecting performance determinants (Table 5). Furthermore, the distribution and progression of interface failure are characterized through uncertainty analysis, and key influencing factors are identified.

**Figure 17:**
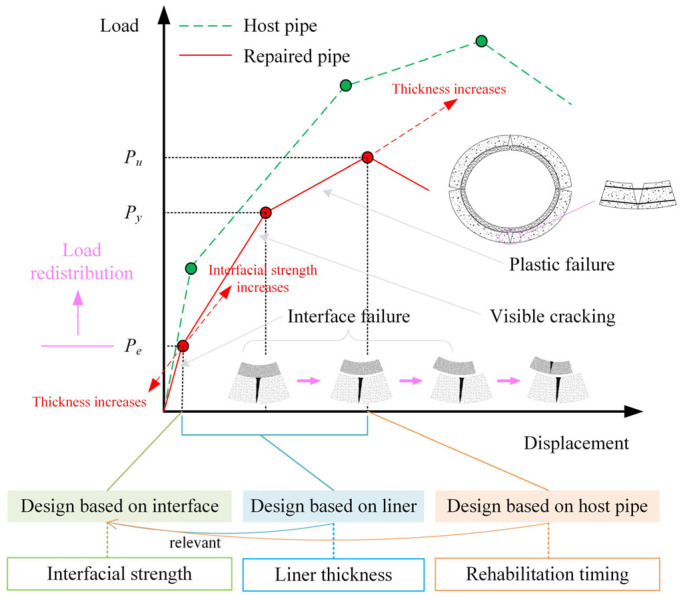
Failure process and design method of RCPs rehabilitated by mortar lining.

Figure 17 illustrates the failure process of mortar-lined RCPs, highlighting the crucial role of interface. Interface failure leads to accelerated cracking and damage of the repaired pipe. The elastic limit load represents the combined effect of interface failure at all angular orientations. Increasing the elastic limit load delays the load redistribution and ultimately enhances the bearing capacity. Therefore, interface design is a critical component of SAPL design, with an emphasis on improving interfacial strengths rather than increasing liner thickness. For example, if the interfacial shear strength is 4 MPa, the elastic limit load can reach approximately 45 kN/m. To achieve this effect, the interfacial strengths can be improved through methods such as chiseling, installing rebars and applying interfacial agents. Additionally, advanced spraying construction techniques should be employed to control thickness uniformity and reduce interface failure probability. Only after interfacial improvements can the liner thickness be optimally calibrated to balance performance and cost. Since the residual bearing capacity is closely related to the damage extent of the host pipe, the rehabilitation timing becomes an important factor in limit state design. Timelier rehabilitation preserves the integrity of the host pipe, enhancing its post-rehabilitation strength.

Existing fiber-reinforced mortar materials exhibit significantly enhanced tensile strength. However, their full tensile potential remains constrained by weak interfacial bonding between concrete and mortar due to inadequate adhesion. Additionally, the mortar manufacturing process may present notable energy consumption challenges. Another critical challenge is the possibility of fiber detachment in corrosive drainage environments, which may contribute to microfiber pollution in wastewater systems. Future material development efforts should prioritize the balance between interfacial adhesion, mechanical performance, spray ability, and eco-friendly performance to ensure sustainable service.

## 5. Conclusions

To investigate the failure behavior of damaged RCPs rehabilitated with fiber-reinforced mortar lining, we developed a new model to describe the failure behavior. This model was applied to the results of the TEB tests, revealing a common pattern of failure and leading to the following conclusions:

In the load-displacement curves, the elastic limit loads of the repaired pipes are 57% and 39% of the pre-rehabilitation conditions, respectively, while their ultimate loads are 45% and 69%. A pipe with a thick liner reduces the elastic limit load but significantly increases the ultimate load, which is primarily caused by interface failure. The interface failure load can be classified into stripping load and slipping load, corresponding to their respective failure behaviors. Interface failure is mainly influenced by interfacial strengths, with little effect from liner thickness. However, uneven liner thickness can increase the failure probability.

After interface failure, the load is redistributed, with the liner and host pipe each bearing a portion of it. A thick liner improves stiffness and slows cracking, leading to a higher ultimate load. Therefore, priority must be given to enhancing the interface in rehabilitation design, followed by consideration of liner thickness. Load redistribution should be incorporated into thickness optimization strategies. Under the operational conditions examined in this study, a spraying thickness of 49 mm restores the repaired pipe to its original bearing capacity.

## Figures and Tables

**Table 1:** Parameters of the host pipes per 1 m length.

Internal Diameter (mm)	Thickness (mm)	Cage Position	Circular Rebar				Longitudinal Rebar	
			*D_C_* (mm)	*ID_C_* (mm)	Count	Distance (mm)	*D_L_* (mm)	Count
1000	100	Inner	5	1040	25	40	5	12
		Outer	5	1150	18.8	53.1	5	12

*D_C_* and *D_L_* are the diameters of the circular and longitudinal rebars, respectively; *ID_C_* is the inner diameter of the circular rebars.

**Table 2:** Measured mechanical parameters of the concrete and mortar.

Material	Compressive Strength (MPa)	Tensile Strength (MPa)	Flexural Strength (MPa)	Elastic Modulus (GPa)	Standard
Concrete	53.83 (2.71%) ^1^	2.43 (9.27%) ^1^	4.51 (0.13%) ^1^	38.1 (8.01%) ^1^	1. GB/T 50081-2019 [36] 2. JGJ/T 70-2009 [37] 3. HPFRCC [38] 4. GB/T 7897-2008 [39]
Mortar	102.3 (5.19%) ^2^	3.13 (7.23%) ^3^	9.66 (1.02%) ^4^	43.1 (2.95%) ^4^	

Numbers 1–4 indicate referenced standards, with different tests conducted according to different standards.

**Table 3:** Structure performance of pre- and post-rehabilitated RCPs.

Specimen	Elastic Limit Load (kN/m)	Displacement (mm)	Stiffness *EI* (kN·mm)	Yield Load (kN/m)	Displacement (mm)	Ultimate Load (kN/m)	Displacement (mm)
A-34	22.6	0.50	28.19 × 10^8^	52.3	5.62	60.2	14.58
B-45	16.1	0.38	27.34 × 10^8^	52.8	4.88	93.8	17.97
A	39.9	1.31	22.21 × 10^8^	119.4	18.69	133.3	28.88
B	41.3	0.98	14.25 × 10^8^	115.0	17.36	135.3	36.28

**Table 4:** Liner thickness calculation equation.

Thickness Design Model	Calculation Equation	Parameter
Thin tube model	$t=\sqrt[{2.5}]{\left(\frac{q_{t}Lr^{1.5}{\left(1-v^{2}\right)}^{0.75}}{0.807E_{L}}\frac{N}{C}\right)}$	*q_t_* is the external load. *L* is the effective length caused by surface traffic wheels, which is taken as the pipe length here. *r* is the inside radius of the host pipe. *E_L_* is the elastic modulus of the liner. *N* is the safety factor, and *C* is the ovality reduction factor, as defined in ASTM F1216 [45].
Distributed beam model	$t=\sqrt{\left(\frac{0.0744q_{t}r^{2}}{S_{F}}\frac{N}{C}\right)}$	*S_F_* is the flexural strength of the liner.
Bazant–Cao scaling model	$\frac{6q_{t}r}{\pi t^{2}}\frac{N}{C}=B\sigma_{TL}{\left(1+\frac{t}{\lambda_{o}d_{a}}\right)}^{-\frac{1}{2}}$	*λ_o_* and B are the scaling parameters. *d_a_* is the maximum aggregate size. *σ_TL_* is the tensile strength of the liner.
Crack propagation model	$t=\sqrt{\frac{7.0464q_{t}r^{2}}{w_{d}E_{L}}\frac{N}{C}}$	*w_d_* is the design crack width, *w_d_* = 0.0625 in (1.59 mm) or 0.01 in (0.25 mm).

**Table 5:** Relative error between theoretical predictions and measurements of design parameters.

Specimen	Interface Failure Load			Lining Thickness
	Stripping Load (Crown/Invert)	Stripping Load (Springlines)	Slipping Load (Crown/Invert)	
A-34	Advanced SCB model			Modified crack propagation
	-	-	5.6%	14.5%
	Original SCB model			Crack propagation
	-	-	34.9%	38.8%
B-45	Advanced SCB model			Modified crack propagation
	0.9%	3.1%	3.2%	0.2%
	Original SCB model			Crack propagation
	-	-	24.2%	35.3%

## Data Availability

The original contributions presented in this study are included in the article. Further inquiries can be directed to the corresponding author.
